# Management of 14 patients with cornual heterotopic pregnancy following embryo transfer: experience from the past decade

**DOI:** 10.1186/s12958-021-00834-w

**Published:** 2021-10-06

**Authors:** Sichen Li, Mingzhu Cao, Hanyan Liu, Yuxia He, Jianqiao Liu

**Affiliations:** 1 Department of Obstetrics and Gynecology, Center for Reproductive Medicine, Key Laboratory for Major Obstetric Diseases of Guangdong Province, The Third Affiliated Hospital of Guangzhou Medical University, 63 Duobao Road, Guangzhou, Guangdong China; 2 Key Laboratory for Reproductive Medicine of Guangdong Province, The Third Affiliated Hospital of Guangzhou Medical University, Guangzhou, China

**Keywords:** Cornual heterotopic pregnancy, Embryo transfer, Transvaginal embryo reduction, Laparoscopic cornual resection

## Abstract

**Objective:**

There are two major management approach for cornual heterotopic pregnancy, transvaginal cornual embryo reduction with ultrasound guidance, or laparoscopic cornual resection. This no consensus on the optimal management for cornual heterotopic pregnancy. Here, we are trying to determine the optimal management approach for patients with viable cornual heterotopic pregnancy following embryo transfer.

**Methods:**

This is a retrospective cohort study conducted at the locally largest reproductive center of a tertiary hospital**.** A total of 14 women diagnosed as viable cornual heterotopic pregnancy following embryo transfer. Six patients were treated with cornual pregnancy reduction under transvaginal ultrasound guidance without the use of feticide drug (treatment 1), and eight patients were treated with laparoscopic cornual pregnancy resection (treatment 2).

**Results:**

All 14 patients of cornual heterotopic pregnancy following embryo transfer due to fallopian tubal factor, among which, 12 patients had cornual pregnancy occurred in the ipsilateral uterine horn of tubal pathological conditions. Nine (64.29%) showed a history of ectopic pregnancy. Thirteen (92.86%) patients were transferred with two embryos and only one patient had single embryo transferred. Six patients received treatment 1, and 2 (33.33%) had uterine horn rupture and massive bleeding which required emergency laparoscopic surgery for homostasis. No cornual rupture occurred among patients received treatment 2. Each treatment group had one case of spontaneous miscarriage. The remaining 5 cases in treatment 1 group and the remaining 7 cases in treatment 2 group delivered healthy live offspring.

**Conclusion:**

Patients with tubal factors attempting for embryo transfer, especially those aiming for multiple embryos transfer, should be informed with risk of cornual heterotopic pregnancy and the subsequent cornual rupture. Compared with cornual pregnancy reduction under transvaginal ultrasound guidance, laparoscopic cornual resection might be a favorable approach for patients with viable cornual heterotopic pregnancy.

## Introduction

Heterotopic pregnancies refers to the coexistence of two or more implantation locations [1]. Heterotopic pregnancies are extremely rare in natural conceptions, with an incidence of 1 in 30,000 pregnancies [2]. However, due to the wide application of assisted reproductive technologies (ART), especially in vitro fertilization (IVF)/intra-cytoplasmic sperm injection (ICSI) and embryo transfer techniques, the incidence of heterotopic pregnancy has edged up recently. For patients received ART treatment, the incidence of heterotopic pregnancy ranged from 1:100 to 1:500 [3–5]. Among various ectopic pregnancy sites, cornual pregnancy is quite rare, with an estimated incidence of 1 in every 3600 pregnancies following ART [6].

Heterotopic pregnancy is often considered as a tricky type of ectopic pregnancy, and the situation is more complicated for cornual heterotopic pregnancies. One major challenge of the management of cornual heterotopic pregnancy is how to suppress the growth of cornual gestation and prevent life-threatening uterine cornual rupture, without interruption to the growing intrauterine fetus [1]. Two main therapeutic strategies were reported in literatures and used in clinical practice, gestational sac removal by cornual incision under laparoscopy/laparotomy, and transvaginal embryo reduction under sonography monitoring [1, 7, 8]. However, only a few case reports proposed the above management strategies and the comparison of prognosis between the two treatment options are even rarer. Up to now, no consensus on the ideal treatment options for cornual heterotopic pregnancy [1, 9], since no sufficient evidences were available to recommend any management strategy.

To the best of our knowledge, no standard management exist for heterotopic cornual pregnancy. In this study, a retrospective cohort analysis was conducted to compare the pregnant outcomes, including live birth rate, miscarriage rate, and cornual rupture rate following the two treatment approaches for patients with cornual heterotopic pregnancies. This study was based on the experience of clinicians in a high-volume reproductive center for more than a decade. The main objective of this study was to determine the optimal treatment options for patients with cornual heterotopic pregnancies following embryo transfer.

## Patients and methods

### Patients’ data inclusion

This was a retrospective cohort study which was conducted at the locally largest reproductive center between January 2008 and December 2018. The study protocol has obtained ethical approval from local ethical committee (Approval number, 2020–004). Clinical data of 17 cornual heterotopic pregnancies following IVF/ICSI and embryo transfer were collected from the electrical database of reproductive center and in-hospital medical archives. Patients met the following criteria were excluded, 1) patients had cornual heterotopic pregnancy through natural conception, 2) patients were confirmed to have ectopic pregnancy at fallopian tube, 3) patients had at least one fetus without fetal heart beat in cornual heterotopic pregnancy. Fourteen cases with cornual and intrauterine heterotopic pregnancies were included in the current study, and all fetuses were confirmed to have cardiac activities under sonography examination.

The diagnosis criteria for cornual heterotopic pregnancy were, 1) at least two gestational sacs were observed, with one gestational sac seen separately and more than 1 cm from the most lateral edge of the intrauterine cavity, 2) less than 5 mm myometrial layer surrounding the gestational sac, and 3) intrauterine gestational sac(s) were also confirm under sonography, 4) all fetuses showing active heart beats.

Clinical data including demographic information, embryo transfer cycle related characteristics, surgical treatment details, reproductive outcomes were reviewed for analysis. Informed consent of each patient had been obtained for the collection and analysis of their clinical data.

### Treatment approaches

Once diagnosed as cornual heterotopic pregnancy, patients were hospitalized immediately. Two treatment approaches, transvaginal ultrasound-guided selective cornual embryo reduction and laparoscopic cornual resection were provided in the current study. Since no ideal treatment approach has been confirmed, and no patient showed any signs of cornual rupture before the initiation of treatment, individual treatment methods were provided based on clinicians’ expertise, patients clinical signs, and patients’ personal choices.

Six patients had transvaginal ultrasound-guided selective cornual embryo reduction (treatment 1) without the use of feticide drug (for instance, methotrexate, MTX and potassium chloride, KCl), which was performed to aspirate fetal heart inside the ectopic cornual gestational sac about 4 to 6 weeks following embryo transfer. Patients were disinfected in the vaginal with povidone-iodine solution. All patients received the procedure without sedation or anesthesia. The intrauterine and cornual gestations were observed and monitored under high-resolution sonography (Voluson P8, GE Healthcare, WI)). The aspirations were conducted using an 18-gauge needle (Wallace 18 G oocyte retrieval set, Smiths Medical, MN) under vacuum and guided real-time by transvaginal ultrasound monitoring. The cornual gestation was observed and oriented with the punctuation line equipped in the sonography. A needle passed through the uterine myometrium and aimed at the corneal gestational sac and fetal heart inside. The cornual gestation fetal heart was then punctured and aspirated with the tip of the needle with the help of the vacuum. The vacuum suction pressure was set to about 150–160 mmHg, and kept constant pressure during the procedures. In case the active fetal heart beat still existed after the fist aspiration, the surgeon would rotate the needle gently and aspirated again. No feticide drugs, including methotrexate and potassium chloride, were allowed to avoid the potential hazards on the intra-uterine gestation. The absence of fetal heart beat inside the cornual gestational sac and presence of active fetal heart beat inside the intra-uterine gestational sac were monitored under the transvaginal sonography for at least 15 min before the completion of the procedure. The fetal heart beats in the cornual gestational sac and intra-uterine gestational sac were detected again at 1-day, 3-day, and 7-day post-operatively.

Eight patients received laparoscopic cornual resection (treatment 2), in which cornual wedge resection were performed once cornual pregnancy was confirmed. The laparoscopic surgeries were perfomed under general anesthesia. The pneumoperitoneum was achived and maintained with carbon dioxide. Three Trocars (Olympus Medical Systems Corp., Tokyo, Japan) were provided with one Trocar placed above the umbilical region, one on the left side and the remaining one on the right side of lower abdomen. The enlarged uterus and distended uterine cornua were observed under the laparoscopy. The fallopian tubes and ovaries were generally observed to be normal in appearances. The distended cornus were than incised with monopolar electrode. The gestation masses were resected and removed completely. Bipolar coagulation was used to stop the bleeding of the resected area. The uterine cornua was further closed with absorbable sutures. The surgical duration were around 40–70 min. No drain tubes were inserted if no active intra-abdominal bleeding were appeared. The removed gestational masses were sent for pathological confirmation. Each one had been hospitalized and monitored for 7 days following operation. All the patients were confirmed to show no further enlargement and active fetal heart beat in cornual areas, and maintaining of live fetuses in intrauterine gestational sacs through sonography examination every other day for one week during hospitalization.

In both procedures, 40 mg/d progesterone (Baiyunshan Pharmaceutical., Guangzhou, China) was provided intramuscularly 2 days before the procedure, and lasted until 5 days following the above procedures. Oral Dydrogesterone (20 mg/d, Abbott Biologicals B.V., the Netherlands) and vaginal suppositories of micronized progesterone (Utrogestan, 400 mg/d, Besins Healthcare, France) were provided until gestational 10 weeks. All patients were followed up with sonography 2-week and 4-week post-operatively to detect the absence of fetal heat beat in the cornual gestation and presence of fetal heart beat in the intra-uterine gestation. All patients were then under the routine obstetrical follow-up until the delivery of live birth offspring.

With in one-week following the both procedures, no patients complained of massive vaginal bleeding or lower abdominal pain. However, two cases had cornual rupture at 12 and 13 days following treatment 1 (gestational 8 and 9 weeks, respectively) before they had a repeated ultrasound examination 2-week following treatment.

### Statistical analysis

All statistical analysis was carried out using SPSS 22.0 (Chicago, Illinois, US). Quantitative data following normal distribution were demonstrated as mean ± SD, and compared using Student *t* test. Quantitative data which were not normally distributed were demonstrated as median and range. Comparisons of those data were based on non-parametric test. Categorical data were presented as number and proportion. Comparisons between categorical variables were performed by the chi-square test and the Fisher’s exact test as appropriate. *P* < 0.05 was considered as statistically significant.

## Results

**Fig. 1:**
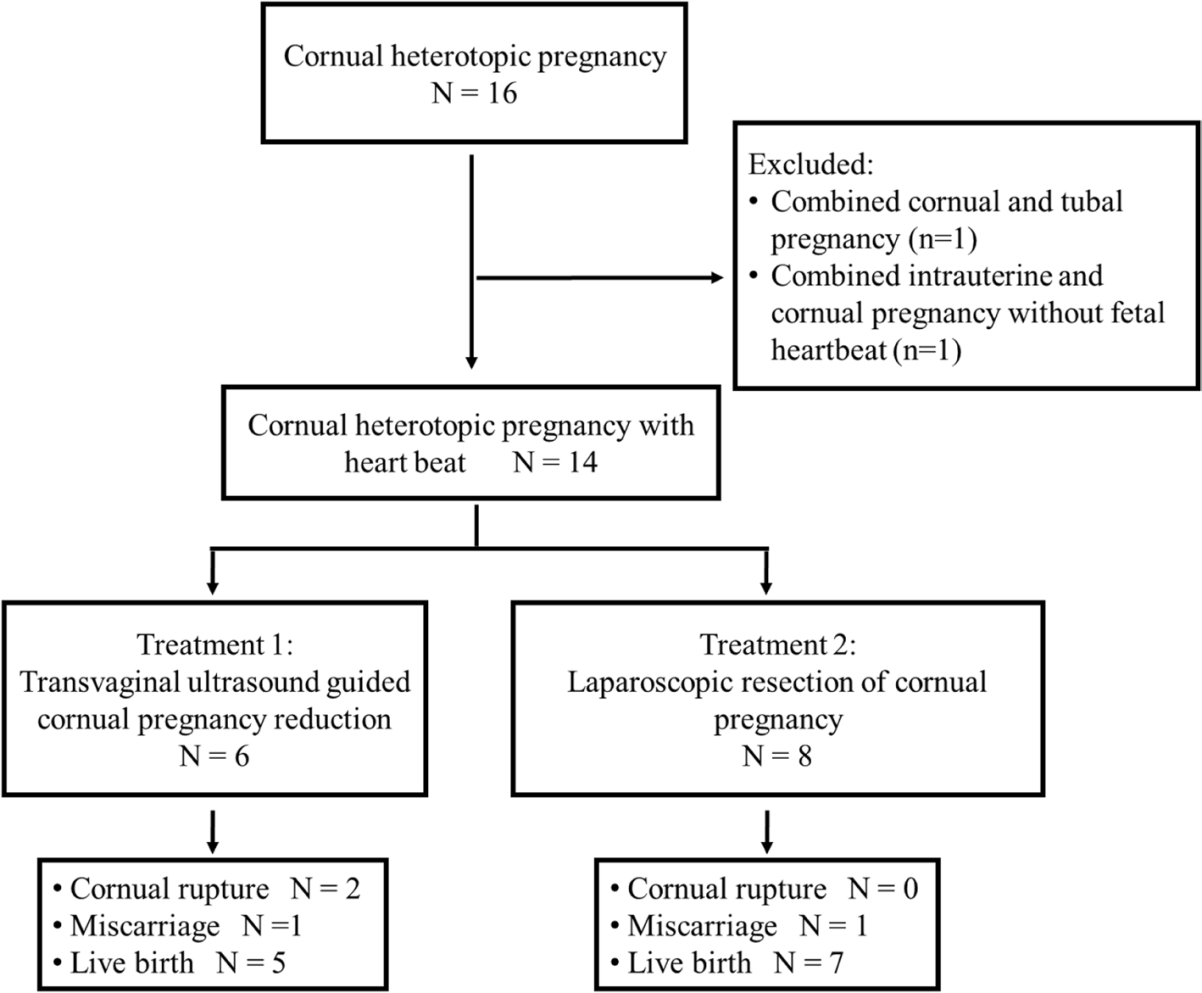
A flow chart of screening and follow-up of study participants

Through reviewing the local databases, 16 cases of cornual heterotopic pregnancies following IVF/ICIS and embryo transfer were reviewed. As clearly demonstrated in Fig. 1, 2 cases were excluded with one case of combined cornual and tubal pregnancy, and one case of combined intrauterine and cornual pregnancy without fetal heartbeat. Among the remaining 14 patients, 6 cases were treated with cornual heterotopic pregnancy reduction under vaginal ultrasound guidance (treatment 1) and 8 cases underwent laparoscopic resection of cornual pregnancy (treatment 2). The included patients were divided into two groups based on the different treatment approaches, treatment 1 (*n* = 6) and treatment 2 (*n* = 8).

The baseline clinical characteristics between the two groups were compared and shown in Table 1. The mean age, body mass index (BMI), gravidity, parity, infertility type, and infertility duration between the two groups showed no significant differences. Even though no differences were detected between the two groups regarding history of ectopic pregnancy, majority of all the patients (9 out of 14 cases, 64.3%) had at least one ectopic pregnancy. Particularly, half of the patients (7 out 14 cases, 50.00%) had multiple ectopic pregnancies. No differences of infertility type were found in patients between the two groups. Interestingly, we did observe that all patients received IVF/ICSI treatment at least partially due to fallopian tube dysfunctions. Nine (64.3%) cases had tubal ligation or excision, among which 7 cases (77.8%) were detected to have the gestational sacs located at the ipsilateral uterine horn of tubal ligation or excision. The remaining 5 cases (35.7%) had no history of fallopian tube surgeries but tubal obstruction or inflammation as determined by hysterosalpingography. Among those 5 cases, 2 cases (40.0%) were confirmed to have the gestational sacs located at the ipsilateral uterine horn of blocked tube and 3 cases (60.0%) at the ipsilateral uterine horn of tubal inflammation.

**Table 1:** Comparisons of baseline clinical features between two treatment approaches

	All (*n* = 14)	Treatment 1 (*n* = 6)	Treatment 2 (*n* = 8)	*P* value
Age (years old)	29.43 ± 2.56	29.88 ± 2.53	28.83 ± 2.71	0.984
BMI (kg/m^2^)	22.08 ± 3.72	21.58 ± 2.95	22.75 ± 4.78	0.162
Gravidity	0 (0–8)	2 (0–8)	1 (0–4)	0.491
Parity	0 (0–1)	0 (0–1)	0 (0–1)	0.573
History of ectopic pregnancy (n/%)				0.491
*N* = 0	5/35.7%	3/50.0%	2/25.0%	
*N* = 1	2/14.3%	1/16.7%	1/12.5%	
*N* = 2	5/35.7%	1/16.7%	4/50.0%	
*N* ≥ 3	2/14.3%	1/16.7%	1/12.5%	
Infertility type				0.245
Primary	4/28.6%	3/50.0%	1/12.5%	
Secondary	10/71.4%	3/50.0%	7/87.5%	
Infertility duration	2.82 ± 1.84	2.81 ± 2.30	2.83 ± 1.17	0.640
Infertility factor				0.365
Tubal factor only	11/78.6%	4/66.7%	7/87.5%	
Mixed factor (Tubal and ovulation)	3/21.4%	2/33.3%	1/12.5%	
Male factor	0/0%	0/0%	0/0%	
Ovulation disorder	0/0%	0/0%	0/0%	

Abbreviations: *BMI* body mass index

Embryo transfer cycle related characteristics between the two treatment groups were compared in Table 2. The cycle number of embryo transfer, fresh or frozen embryo for transfer, ovarian stimulation protocol, and endometrial preparation protocol for freeze-thawed embryo transfer showed no detectable differences between the two groups. In this study, only one case had one embryo transferred, and the remaining 13 (92.86%) cases had two embryos transferred. Cleavage-stage embryos were transferred in 8 cases (57.14%), morula in 1 case (7.14%) and blastocysts (35.71%) in 5 cases. All cases confirmed their diagnosis at around 6.5 gestational weeks with the first sonography examination following embryo transfer.

**Table 2:** Comparisons of embryo transfer cycle characteristics between two treatment approaches

	All (*n* = 14)	Treatment 1 (*n* = 6)	Treatment 2 (*n* = 8)	*P* value
Cycle number of embryo transfer				1.0
*N* = 1	4/28.6%	2/33.3%	2/25.0%	
*N* = 2	6/42.9%	2/33.3%	4/50.0%	
*N* ≥ 3	4/28.6%	2/33.3%	2/25.0%	
Cycle type				0.277
Fresh embryo transfer	6/42.9%	4/66.7%	2/25.0%	
Frozen embryo transfer	8/57.1%	2/33.3%	6/75.0%	
Ovulation stimulation protocol				1.00
GnRHa protocol	10/71.4%	4/66.7%	6/75.0%	
GnRHant protocol	4/28.6%	2/33.3%	2/25.0%	
Endometrial preparation protocol in frozen embryo transfer				0.464
NC	2/25.0%	1/16.7%	1/50.0%	
HRT	6/75.0%	5/83.3%	1/50.0%	
No. of embryo for transfer				0.662
*n* = 1	1/7.1%	1/16.7%	0/0%	
*n* = 2	13/92.9%	5/83.3%	8/100%	
*n* = 3	0/0%	0/0%	0/0%	
Embryo stage				0.755
Cleavage	8/57.1%	4/66.7%	4/50.0%	
Morula	1/7.1%	0/0%	1/12.5%	
Blastocyst	5/35.7%	2/33.3%	3/37.5%	
Gestational age at diagnosis (wk)	6.5 ± 0.55	6.5 ± 0.53	6.5 ± 0.55	1.0

Abbreviation: *GnRHa* gonadotropin releasing hormone agonist, *GnRHant* gonadotropin releasing hormone antagonist, *NC* natural cycle, *HRT* hormone replacement therapy

The incidences of cornual heterotopic pregnancy were further evaluated based on the embryo transfer cycle related characteristics. As clearly presented in Table 3, among those women with clinical pregnancies, the incidence of cornual heterotopic pregnancy was a little lower in fresh embryo transfer (1/2039) than in freeze-thawed embryo transfer cycle (1/1553). More importantly, among women with clinical pregnancies, the incidence of cornual heterotopic pregnancies in patients with two embryo transferred (1/1500) almost doubled compared with patients with only one embryo transferred (1/2850). Regarding the stages of embryo, cleavage embryos (1/1841) had similar occurrence of cornual heterotopic pregnancy, as transfer of morula resulted in 1/1039 incidence, and blastocyst resulted in 1/1533 incidence of cornual heterotopic pregnancies.

**Table 3:** Incidence of cornual heterotopic pregnancy and related cycle characteristics

	Women with clinical pregnancies	Cornual heterotopic pregnancies	Incidence of cornual heterotopic pregnancies
All cycles with embryo transfer	24,659	14	1/1761
Cycle type			
Fresh embryo transfer	12,234	6	1/2039
Frozen embryo transfer	12,425	8	1/1553
No. of embryo for transfer			
*n* = 1	2850	1	1/2850
*n* = 2	19,498	13	1/1500
Embryo stage			
Cleavage	14,731	8	1/1841
Morula	1039	1	1/1039
Blastocyst	7665	5	1/1533

The reproductive outcomes were compared between patients received two treatment approaches (Table 4). No patient had cornual rupture after received laparoscopic resection of cornual pregnancy. However, 2 (33.33%) out of 6 patients who were treated with cornual pregnancy reduction under transvaginal ultrasound guidance, had uterine horn rupture and massive bleeding post-operatively. Both of them required emergency laparoscopic surgery to repair ruptured cornua. There was one case with spontaneous miscarriage following each treatment approach. The remaining cases all had live birth, and the live birth rates were similar between the two groups (83.3% vs. 87.50%). The mode of delivery and newborns’ birth weights also showed no differences as presented in Table 4.

**Table 4:** Comparisons of reproductive outcomes between two treatment approaches

	All (*n* = 14)	Treatment 1 (*n* = 6)	Treatment 2 (*n* = 8)	*P* value
Rupture after treatment (n/%)	2/14.29%	2/33.33%	0	0.165
Miscarriage rate (n/%)	2/14.29%	1/16.67%	1/12.5%	1.0
Live birth rate (n/%)	12/85.71%	5/83.33%	7/87.50%	1.0
Mode of delivery (n/%)				0.417
Vaginal	1/7.14%	1/20.00%	0/0%	
Cesarean	11/92.86%	4/80.00%	7/100%	
Newborns’ birth weight (kg)	2.78 ± 0.41	2.57 ± 0.29	2.91 ± 0.44	0.646

## Discussion

The optimal treatment approach for cornual heterotopic pregnancy remains controversial. In this study, we performed a retrospective analysis of 14 patients diagnosed with cornual heterotopic pregnancy in the past decade. Patients received either cornual pregnancy resection under laparoscopy or transvaginal fetal reduction under guidance of sonography. Here, based on the results of this study, we would recommend the laparoscopic cornual resection for the safe management of patients with cornual heterotopic pregnancy with visible fetal heartbeat.

This study showed that all patients with cornual heterotopic pregnancy had fallopian tube pathological conditions and majority of them had a history of ectopic pregnancy. More interestingly, 12 out of 14 cases present the cornual pregnancies at the same side of tubal pathological conditions, regardless of whether the salpingectomy was performed. This phenomenon indicated a close relationship between tubal dysfunction and occurrence of cornual pregnancy. Even after the excision or ligation of fallopian tube, the predisposed risk of cornual pregnancy remained. This proposal is consistent with other researchers’ opinions [1, 10], suggesting that tubal injury and history of salpingectomy are major risk factors for cornual pregnancy. In fact, the risk of cornual pregnancy might be raised with the use of ART after bilateral salpingectomy [1]. The possibilities of cornual pregnancy even after the excision and ligation of fallopian tube should be informed prior to the initiation of ART cycles.

Patients with two embryos transferred showed almost doubled incidence of cornual heterotopic pregnancy as compared to those with only one embryo transferred. The risk of cornual heterotopic pregnancy appears to be closely related with multiple embryos for transfer [11]. Some researchers proposed that no more than two embryos should be transferred in patients with precursors to reduce the risk of ectopic and cornual pregnancy [12]. Based on the results of this study, we should be more prudent on the above proposal. Single embryo transfer for patients with high risk of cornual heterotopic pregnancy might be a preference.

Due to the scarcity of the disease, no sufficient data are available regarding the ideal treatment methods. Treatment options of cornual heterotopic pregnancy include expectant management with close monitoring, medical, and surgical treatments. Expectant treatment might be a choice if the ectopic embryo showed no heart beat and a limited craniocaudal length [13]. However, the experience and reports on expectant management are very limited with only few case reports [13, 14].

One review suggested medical therapy for patients without signs of cornual rupture, resulted in a similar prognosis as surgical treatment (live birth rates, 50% vs. 60.9%) [1]. However, this conclusion should be drawn with prudence. First, this comparison was made between patients at different gestational ages. Medical treatment was provide for patients diagnosed at an average gestational 7 weeks with no signs of cornual rupture, whereas surgical treatment was provided for patients at gestational 11.5 weeks with 73.9% patients already showing cornual rupture. Second, the medical treatment was performed by administrating KCl or MTX in the gestational sac or fetal heart. It is worth mentioning that the miscarriage rate was notably higher in patients received medical treatment (5/10, 50%) compared to those had surgical treatment (3/23, 13%). The high miscarriage rate of remaining intrauterine gestation might partially due to the use of KCl and MTX. For heterotopic pregnancy, how to maintain the viable intrauterine pregnancy is another critical aspect to be considered. Even the local injection of KCl or MTX cannot completely avoid the potential impact on intrauterine pregnancy. In the present study, to minimize the interruption of the intrauterine fetus growth, only mechanically aspiration was provided, instead of injection of feticide medications. However, rupture of uterine horns occurred in two out of six cases received above treatment, although the fetuses of cornual sac were completely aspirated and removed. This might indicated that trophoblast attached to the uterine myometrium may continue to proliferate even after the fetus had been removed, which may cause cornual rupture and subsequent massive bleeding. A close monitoring of those patients following transvaginal embryo resection is highly required.

Laparoscopic cornual resection proved to be an easy and safe technique, with low maternal morbidity and insignificant blood loss [15]. One retrospective analysis of 14 patients indicated that laparoscopic cornuostomy or cornual repair appeared to be an effective treatment for cornual heterotopic pregnancy, including cornual ruptured ones [15]. Habana [1] reviewed 37 cases of cornual heterotopic pregnancy and concluded that patients who underwent surgery with a lower miscarriage rate and a higher live birth rate compared with the medical treatment group. Indeed, surgical resection of uterine horns was safely performed in the current study with no rupture of uterine cornua. However, patients still should be informed of the raised risk of uterine horn rupture with prolonged gestational weeks, since the reported average gestational age of cornual rupture was 12 weeks [1].

Miscarriage rate of heterotopic pregnancy following treatment was around 6 to 33% [16]. Here, in this study, the overall miscarriage rate was 14.29%, and a similar miscarriage rate was observed between the two treatment approaches. According to Na [16], in those with heterotopic pregnancies, gestational age at treatment is the only risk factor for miscarriage, regardless of treatment approaches. Indeed, in this study, the early diagnosis (gestational 6.5 weeks) and immediate treatment might contribute to the low miscarriage rate.

This study presented a relatively large number of cornual heterotopic pregnancies with active fetal heartbeats following embryo transfer. The currently available treatment approaches were compared to determine an optimal strategy. However, several limitations should be noted. The first one is the potential bias from retrospective design of the study. Next, the sample size is still quite limited. However, clinical trials with a larger sample size comparing the efficacy of treatment approaches are very difficult due to its rarity. Multi-center collaboration in the future study might be helpful.

In conclusion, patients with tubal pathological conditions and multiple embryos transferred have higher risk of developing cornual heterotopic pregnancy. Compared with transvaginal cornual embryo reduction under ultrasound monitoring, fewer cornual rupture occurred in laparoscopic cornual resection. Similar live birth rate and miscarriage rate were observed between patients received either treatment approach. Consequently, patients attempt to conceive through ART, especially those with tubal factor and requiring multiple embryos transfer, should be informed of the risk of cornual heterotopic pregnancy and the following complications. Once cornual heterotopic pregnancy occurred, we suggest that the laparoscopic cornual resection might be a better treatment approach for patients with cornual heterotopic pregnancy and active fetal heartbeat following embryo transfer.

## Data Availability

The datasets used and/or analyzed during the current study are available on reasonable request.
